# Impact of late embryonic and early fetal mortality on productivity of beef cows

**DOI:** 10.1093/tas/txaf071

**Published:** 2025-05-29

**Authors:** Lucas Melo Gonçalves, Samir Burato, Lucas Neira, Kelsey Harvey, Saulo Menegatti Zoca, Vitor Rodrigues Gomes Mercadante, Pedro Levy Piza Fontes

**Affiliations:** University of Georgia, Department of Animal and Dairy Science, Athens, GA 30602, USA; University of Georgia, Department of Animal and Dairy Science, Athens, GA 30602, USA; University of Illinois, Dixon Springs Agriculture Center, Simpson, IL 62985, USA; Mississippi State University, Prairie Research Unit, Prairie, MS 39756, USA; University of Tennessee, Department of Animal Science, Knoxville, TN 37996, USA; Virginia Tech, Department of Animal and Poultry Sciences, Blacksburg, VA 24061, USA; University of Georgia, Department of Animal and Dairy Science, Athens, GA 30602, USA

**Keywords:** Beef cattle, calving distribution, cow productivity, embryonic mortality, pregnancy loss

## Abstract

Although late embryonic/early fetal mortality (LEEFM) has been extensively researched in the context of reproductive efficiency, its long-term impact on cow productivity over a complete production cycle is poorly described in the scientific literature. This study used a prospective cohort design to evaluate the impact of late LEEFM on the productivity of beef cows. Postpartum cows (n = 2204) were exposed to a fixed-timed artificial insemination (FTAI) protocol followed by natural service breeding for the remainder of the breeding season. Pregnancy status was assessed 28 to 35 and 90 to 115 d after FTAI. Based on pregnancy status, cows were categorized into 1 of 3 cohorts: 1) Cows diagnosed as pregnant to FTAI on both pregnancy diagnoses were considered to have maintained their FTAI pregnancies (MAINT). 2) Cows diagnosed as non-pregnant to FTAI on the first diagnosis were classified as non-pregnant to FTAI (NP). 3) Cows that became pregnant to FTAI and were not pregnant to FTAI at the final pregnancy diagnosis were classified as having experienced LEEFM. Late embryonic and early fetal mortality cows had decreased final pregnancy rates (*P* < 0.01), calving rates (*P* < 0.01), and weaning rates (*P* < 0.01) compared with both MAINT and NP cows. In the subsequent year, LEEFM cows calved later and weaned lighter calves compared with MAINT and NP (*P* < 0.01). In addition, NP and LEEFM also had decreased (*P* ≤ 0.03) pregnancy rates to FTAI and final pregnancy rates in the subsequent year compared with MAINT cows. These results indicate that cows experiencing LEEFM exhibited reduced overall performance not only compared to those that maintained their FTAI pregnancy but also to cows that failed to conceive to FTAI, underscoring the detrimental impact of LEEFM on cow productivity.

## Teaser

Cows undergoing late embryonic/early fetal mortality (LEEFM) after fixed-time artificial insemination (FTAI) are less likely to become pregnant within a controlled breeding season and produce lighter calves compared with cows that maintained their FTAI or cows that failed to conceive to FTAI.

## INTRODUCTION

Most of the income in a cow-calf operation is determined by the number of calves sold at weaning (Ramsey et al., 2005) and reproductive efficiency is the main factor influencing weaning rates in the beef cattle industry (Dill et al., 2015). Timing of conception within the breeding season also impacts herd productivity. Cows that conceive in the beginning of the breeding season calve early in the calving season and wean heavier calves compared with cows that conceive later in the breeding season (Funston et al., 2012; Cushman et al., 2013). In addition, male calves that are born in the first 21 d of the calving season produce heavier carcasses with greater marbling scores and have increased carcass value compared with calves born later in the calving season (Funston et al., 2012). Calving distribution also influences replacement heifer productivity. Heifers born early in the calving season are older and heavier at the beginning of their first breeding season compared with heifers that are born later in the calving season (Funston et al., 2012). Moreover, a greater proportion of replacement heifers born early in the calving season are cycling in the beginning of their first breeding season, leading to greater subsequent pregnancy rates (Funston et al., 2012; Credille et al., 2023). Cows that conceive earlier also have a greater interval between calving and the beginning of the subsequent breeding season, increasing their ability to become pregnant and remain in the herd when compared to cows that conceived later in the season (Stevenson et al., 2003; Cushman et al., 2013).

Pregnancy loss is a major factor that negatively impacts reproductive efficiency in beef and dairy herds (Santos et al., 2004; Ealy, 2020; Mercadante et al., 2020), with cattle experiencing pregnancy loss in different periods of gestation (Wiltbank et al., 2016). In the beef cattle literature, pregnancy loss within the first 28 d of gestation is commonly referred to as early embryonic mortality (EEM; Smith et al., 2022). Pregnancy losses occurring between days 28 and 100 of gestation are commonly defined as late embryonic and early fetal mortality (LEEFM). In a recent meta-analysis, Reese et al., (2020) reported a 5.8% prevalence of LEEFM in beef herds, ranging between 4.8% and 6.9% depending on the study. Factors known to influence to the prevalence of LEEFM include parity (Consentini et al., 2023), expression of estrus (Pereira et al., 2016), temperament (Brandão and Cooke, 2021), health status (Aono et al., 2013; Sanhueza et al., 2013), and the use of assisted reproductive technologies (Ealy et al., 2019; Hansen, 2020). Although LEEFM is less prevalent compared with EEM, Diskin and colleagues (2011) suggested that LEEFM may still reduce herd productivity by preventing cows from rebreeding within the same breeding season. Nevertheless, the current literature lacks a thorough characterization of the impact of LEEFM on beef cow herd productivity. Therefore, the objective of this study was to characterize the impact of LEEFM on the performance of beef cows exposed to fixed-time artificial insemination (FTAI). We hypothesized that cows experiencing LEEFM have decreased pregnancy rates at the end of the breeding season and calve later in the subsequent year compared with cows that maintain their FTAI pregnancy and cows that fail to become pregnant to FTAI. In addition, we hypothesized that differences in calving distribution in cows experiencing LEEFM lead to a decrease in subsequent calf performance and cow fertility.

## MATERIALS AND METHODS

All cows were handled in accordance with procedures approved by the University of Georgia’s Animal Care and Use Committee (A2020 02-002-Y3-A0).

### Animals, Fixed-time Artificial Insemination, and Breeding Season Management

The present observational study followed a prospective cohort design utilizing data from 2204 primiparous (n = 362) and multiparous (n = 1,842) *Bos taurus* beef cows (age: 5.5 ± 3.0; days postpartum: 77.6 ± 18.4; body condition score: 5.5 ± 0.8 using a 1 to 9 scale; Wagner et al., 1988) from six locations across four states in the Southeastern United States: Georgia (n = 605), Mississippi (n = 179), Tennessee (n = 146), and Virginia (n = 1,274). Cows were exposed to either a spring (n = 1,496) or fall (n = 708) breeding season, and all herds were managed on a grazing-based system throughout the breeding period. Grazing systems varied between locations and were comprised of bermudagrass (*Cynodon dactylon*) or fescue (*Schedonorus arundinaceus*). Each location had multiple subgroups of cows, and the size of subgroups varied depending on the management practices of each location.

Data collection started at the beginning of the breeding season of year one and lasted until calves produced during the year one breeding season were weaned the following year (year two). In year one, cows were exposed to two ovulation synchronization protocols, which included the 7-day CO-Synch + CIDR (Larson et al., 2006) or the 7&7 Synch protocols (Oosthuizen et al., 2020; Andersen et al., 2021) depending on the location. Cows exposed to the 7-day CO-Synch + CIDR protocol (n = 1,660) received a 100-μg injection of gonadotropin-releasing hormone (GnRH; 100 μg im; Factrel; gonadorelin hydrochloride; Zoetis Animal Health, Parssipany, NJ) and a controlled intravaginal drug release (CIDR) insert (1.38 g of progesterone; EAZIBREED CIDR; Zoetis Animal Health) on day-10. Prostaglandin F_2α_ (PGF; 25 mg im; Lutalyse HighCon; dinoprost tromethamine; Zoetis Animal Health) was administered upon CIDR removal on day -3, and cows were then exposed to FTAI concurrently with a second GnRH injection 60 to 66 h after CIDR removal (day 0). Cows exposed to the 7&7-Synch protocol (n = 528) received a similar synchronization procedure to cows exposed to the 7-d CO-Synch + CIDR, except an injection of PGF and the CIDR insert were administered on day -17. Beginning 10 to 14 days after FTAI, cows were exposed to cleanup bulls for 53 to 72 days at a bull to cow ratio ≤ 1:25. Hence, the length of the breeding seasons ranged from 67 to 86 d depending on the location. All bulls were classified as satisfactory potential breeders after a breeding soundness evaluation was performed following Society for Theriogenology guidelines (Koziol and Armstrong, 2018).

### Ultrasonography and Pregnancy Loss Classification

Transrectal ultrasonography (Sonoscape S8EXP; SonoScape Medical Corp, Schenzhen, GD, China or EVOII; E.I. Medical Imaging, Loveland, CO, USA) was performed 28 to 35 days after FTAI to determine pregnancy status. Cows were classified as pregnant based on the presence of fluid in the uterine lumen and a conceptus consistent with a 28- to 35-day pregnancy. Cows with evidence of placental detachment, loss of fetal heartbeat, decreased fluid accumulation in the uterine lumen, or abnormal echogenicity of uterine fluid in the first pregnancy diagnosis were classified as non-pregnant. A second pregnancy diagnosis was performed using transrectal ultrasonography within 115 days after FTAI, allowing final pregnancy rates and fetal age to be determined based on fetal morphometries (Fontes et al., 2019). Fetal age was then utilized to distinguish pregnancies generated via FTAI from natural service pregnancies.

Cows were then classified into three different pregnancy status cohorts: 1) Cows that were diagnosed as pregnant to FTAI on both pregnancy diagnoses were classified as having maintained (MAINT) their FTAI pregnancies. 2) Cows diagnosed as non-pregnant to FTAI on the first diagnosis were classified as non-pregnant to FTAI (NP), regardless of their final pregnancy status at the end of the breeding season. 3) Cows that became pregnant to FTAI and were non-pregnant at the time of final pregnancy diagnosis were classified as having experienced LEEFM. Similarly, cows that were pregnant to FTAI in the first pregnancy diagnosis but were pregnant by natural service in the final pregnancy diagnosis were also classified as having experienced LEEFM. After the final pregnancy diagnosis, cows that failed to conceive within the breeding season were culled from the herds, whereas cows that conceived within the breeding season, regardless of their pregnancy status classification described previously, were maintained in the herd until the calf that was a product of that breeding season (year one) was weaned in year two.

Calving date and birth weight were recorded within 24 h from each calving event, with birth weight measured using a hanging analog scale. Calving dates were utilized to evaluate calving distribution based on the average calving day and percentage of calves born within the first, second, or third 21-d intervals of the calving season. The first day of the calving season was considered the day in which the 1^st^ live calf was born in each location. Within location, all calves were weaned on the same day. Calves were weaned at 140 to 286 day of age, and calf weaning weight was recorded at the same day in which calves were weaned. Adjusted weaning weights were also assessed using a 205-day approach, where adjusted weaning weights = birth weight + [205 × (average daily gain between birth and weaning)]. Dam age adjustments were not performed because dam age was not available for all cows. Cows that successfully calved after the year one breeding season were exposed to a subsequent breeding season in year two.

Data from year two breeding season were assessed to evaluate the long-term consequences of pregnancy status classification from year one on pregnancy rates in year two. Breeding season management in year two was performed using the same approach described for year one; however, the timing of pregnancy diagnosis in year two did not allow accurate diagnosis of pregnancy loss. Consequently, only pregnancy rates to AI and final pregnancy rates at the end of the breeding season were evaluated in year two.

### Statistical Analysis

All data analyses were conducted using SAS statistical package (version 9.4; SAS/STAT, SAS Inst. Inc., Cary, NC, USA). Binary response variables were analyzed using the GLIMMIX procedure, while continuous response variables were assessed using the MIXED procedure of SAS. Binary response variables were analyzed using a binary distribution with the logit function, whereas continuous variables were analyzed assuming a Gaussian distribution. Normality of residuals for continuous variables was determined based on the Shapiro-Wilk test, which was performed using the UNIVARIATE procedure of SAS.

Models for both binary and continuous variables included the fixed effects of pregnancy status classification (MAINT, NP, and LEEFM), parity (primiparous or multiparous), and their respective interaction. Moreover, models included the random effect of location. A survival analysis was performed employing the LIFETEST procedure to characterize the effect of pregnancy status classification on cumulative calving events. Survival curves were compared using the Wilcoxon test. Results are presented as least-square means and standard error of the means unless otherwise stated. Significance was defined at *P* ≤ 0.05. Tukey adjusted *P-*values are reported for all least square means differences comparisons to minimize chances of type I error.

## RESULTS

### Impact of Pregnancy Status on First-Year Pregnancy Outcomes

**Figure 1.**
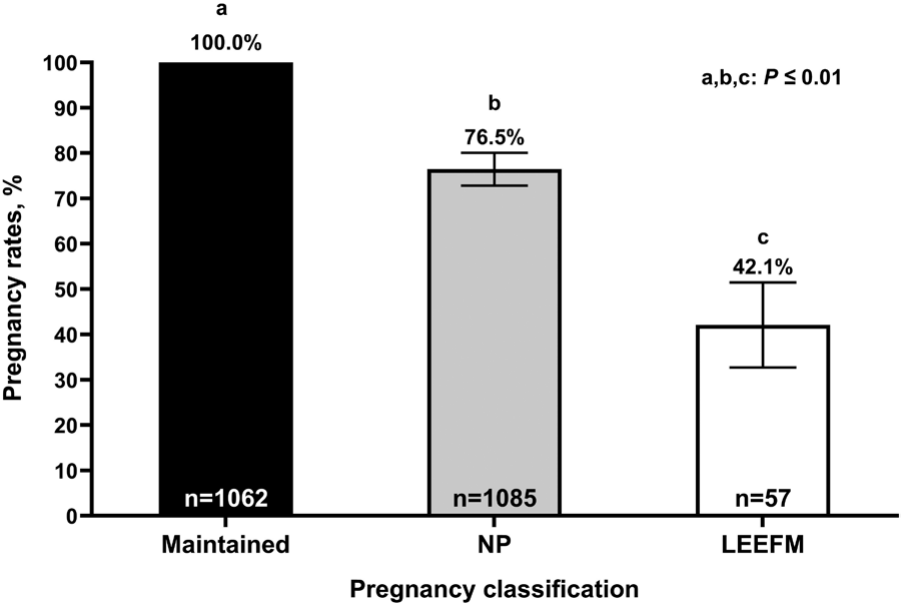
Year one final pregnancy rates in beef cows exposed to fixed-time artificial insemination (FTAI) followed by natural service and categorized by pregnancy status at the final diagnosis. MAINT: Maintained FTAI pregnancy. NP: Non-pregnant to FTAI. LEEFM: Cows classified as undergoing late embryonic/early fetal mortality. ^a,b,c^ Uncommon superscripts indicate statistical differences (*P* ≤ 0.01).

Data describing the pregnancy outcomes in year 1 for each herd enrolled in this study is summarized in Table 1. Overall pregnancy rates to FTAI were 50.8% (1119/2204), whereas final pregnancy rates were 88.3% (1947/2204). Moreover, pregnancy rates to FTAI and final pregnancy rates ranged from 40.8% to 64.0% and 83.4% to 96.6% between locations, respectively. Pregnancy loss between the first and second pregnancy diagnosis occurred in 2.6% (57/2204), which represented 5.1% (57/1119) of the cows that conceived to FTAI. Therefore, a total of 1,062, 1,085, and 57 cows were classified as MAINT, NP, and LEEFM, respectively. Figure 1 describes final pregnancy outcomes in year one based on the pregnancy status cohorts. There was a pregnancy status effect (*P* = 0.01), where MAINT cows had greater final pregnancy rates compared to NP and LEEFM (*P *< 0.01). In addition, final pregnancy rates were also greater (*P* = 0.01) in NP compared with LEEFM cows. There was a parity effect (*P *= 0.01) where multiparous cows had greater pregnancy rates compared with primiparous (91.5 ± 1.53% and 82.1 ± 3.31%, respectively). There was no interaction between parity and pregnancy status on the final pregnancy rates (*P* = 0.89).

**Table 1.** Descriptive data by location enrolled in this study on the first year.

Location	State	Season	N^o^ of cows	DPP^1^	Age	PR/FTAI, % (n/n)^2^	Preg loss AI,% (n/n)^3^	Preg loss herd, % (n/n)^4^	PR/Total, % (n/n)^5^
A	GA	Spring	153	89.5 ± 16.1	4.8 ± 3.0	53.6 (82/153)	6.1 (5/82)	3.3 (5/153)	84.3 (129/153)
B	GA	Spring	54	84.7 ± 28.2	3.3 ± 2.2	53.7 (29/54)	6.9 (2/29)	3.7 (2/54)	94.4 (51/54)
C	MS	Fall	179	78.2 ± 15.5	5.0 ± 2.9	56.4 (101/179)	4.0 (4/101)	2.2 (4/179)	96.6 (173/179)
D	TN	Fall	146	86.6 ± 15.0	5.9 ± 2.8	61.6 (90/146)	6.7 (6/90)	4.1 (6/146)	89.0 (130/146)
E	GA	Spring	197	71.4 ± 23.8	5.3 ± 2.9	64.0 (126/197)	4.8 (6/126)	3.0 (6/197)	90.4 (178/197)
F	GA	Spring	201	78.0 ± 21.4	-	54.2 (109/201)	9.2 (10/109)	5.0 (10/201)	87.1 (175/201)
G	VA	Spring	745	75.7 ± 16.7	5.8 ± 3.1	40.8 (304/745)	3.9 (12/304)	1.6 (12/745)	85.5 (637/745)
H	VA	Fall	529	75.3 ± 15.5	5.8 ± 2.9	52.6 (278/529)	4.3 (12/278)	2.3 (12/529)	89.6 (474/529)
Overall	-	-	2204	77.6 ± 18.4	5.5 ± 3.0	50.8 (1119/2204)	5.1 (57/1119)	2.6 (57/2204)	88.3 (1947/2204)

^1^DPP: Days postpartum at the time of timed artificial insemination (FTAI).

^2^Pregnancy rates to FTAI at each location.

^3^Pregnancy loss occurring between the first (day 28 to 35) and second (<115 days) pregnancy diagnoses, estimated as the proportion of cows that were initially pregnant.

^4^Pregnancy loss occurring between the first (day 28 to 35) and second (< 115 days) pregnancy diagnoses, estimated as the proportion of pregnancy loss relative to the total number of cows in the herd exposed to the breeding season.

^5^Final pregnancy rates at each location.

### Impact of Pregnancy Status on Calving Outcomes

Overall calving rate was 84.4% (1860/2204), ranging from 80.4% to 95.0% between locations. The impact of pregnancy classifications on calving outcomes is summarized in Table 2. A greater proportion of MAINT cows calved compared with both NP (*P *< 0.01) and LEEFM (*P *< 0.01). Moreover, a greater (*P* = 0.01) proportion of NP cows calved compared with LEEFM cows. There was also an effect of pregnancy status (*P* ≤ 0.05) on the percentage of cows calving in the 1^st^, 2^nd^, and 3^rd^ calving periods. During the 1^st^ 21-d calving period, a greater (*P *= 0.01) percentage of MAINT cows calved compared to NP, and no cows classified as LEEFM calved during this period. Alternatively, a greater proportion of NP (*P *< 0.01) and LEEFM (*P *< 0.01) cows calved during the 2^nd^ period compared with MAINT cows, but no differences (*P *= 0.50) were observed between NP and LEEFM. During the 3^rd^ period, no cows classified as MAINT calved and a greater (*P* = 0.05) proportion of LEEFM calved compared with NP. There was no effect of parity (*P *≥ 0.16) or interaction between parity and pregnancy status on the percentage of cows calving within each 21-d calving period (*P *≥ 0.06).

**Table 2.** Comparative calving data between cows that conceived or did not conceive to fixed-time artificial insemination (FTAI) and cows that experienced late embryonic/early fetal mortality (LEEFM).

Item	Pregnancy status^1^			SEM	*P*-value		
	Maintained	Non-pregnant	LEEFM		Status	Parity	Status × Parity
Calving rate^2^, %	97.5^a^	72.2^b^	40.3^c^	9.31	<0.01	0.16	0.20
Calving period^3^, %							
1^st^ period	96.3^a^	9.6^b^	0.0^c^	3.00	0.01	0.56	0.06
2^nd^ period	3.9^a^	55.7^b^	39.9^b^	7.16	<0.01	0.83	0.45
3^rd^ period	0.0^a^	31.6^b^	59.8^c^	13.47	0.05	0.64	0.32
Calving day^4^					<0.01	0.69	<0.01
Multiparous	11.4^a^	35.2^b,x^	50.9^c^	2.89			
Primiparous	10.9^a^	40.9^b,y^	48.3^b^	5.95			

^1^Cows were classified by pregnancy status in year one. MAINT: Maintained FTAI pregnancy; NP: Non-pregnant to FTAI; LEEFM: Late embryonic/early fetal mortality.

^2^Calving rate was calculated as the percentage of cows that calved relative to the total number of cows exposed to the breeding season.

^3^1^st^ Period represented the first 21 days of the calving season. 2^nd^ period ranged from 22 to 42 days after the beginning of the calving season. 3^rd^ period: ranged from 43 days after the beginning of the calving season until the end of the calving season.

^4^Average calving day within the calving season.

^a,b,c^Within a row represents statistical difference between pregnancy status (*P* ≤ 0.05).

^x,y^Within a column represents statistical difference between parity (multiparous or primiparous) within the same pregnancy status (*P* ≤ 0.05).

**Figure 2.**
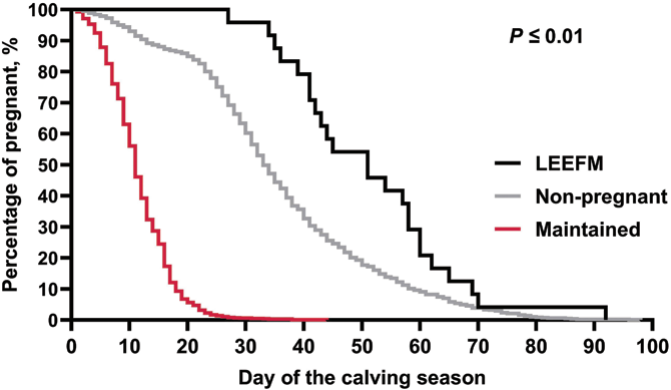
Survival analysis of the percentage of pregnant cows throughout the calving season. MAINT: Maintained FTAI pregnancy. NP: Non-pregnant to FTAI. LEEFM: Late embryonic/early fetal mortality. Log-Rank test: *P* ≤ 0.01.

A pregnancy status × parity interaction was observed (*P* = 0.01) for average calving day of the calving season. Within multiparous cows, LEEFM cows calved later than MAINT and NP (*P* < 0.01), whereas MAINT cows calved earlier (*P* = 0.01) compared with NP cows. In primiparous cows, MAINT cows calved earlier than NP and LEEFM (*P* < 0.01); however, NP did not differ (*P* = 0.81) from LEEFM. Within cows classified as non-pregnant, multiparous cows calved earlier (*P* = 0.01) compared with primiparous. Similar results were observed in the survival analysis (Figure 2), where cows experiencing LEEFM calved later compared with MAINT (*P* < 0.01) and NP cows (*P* < 0.01). Moreover, NP calved later (*P* = 0.01) compared with MAINT cows.

### Impact of Pregnancy Status on Weaning Outcomes

In this present study, 81% (1788/2204) of cows successfully weaned a calf in year 2, and the average calf weaning weight was 246.4 ± 47.4 kg (mean ± SD). Table 3 summarizes the results for response variables collected at weaning. A pregnancy status × parity interaction was observed (*P* = 0.02) for weaning rates. Within multiparous, LEEFM cows had a decreased weaning rate compared with MAINT (*P* < 0.01) and NP cows (*P* < 0.01), and NP had a decreased (*P* = 0.01) weaning rate compared with MAINT cows. Within primiparous, LEEFM cows had a decreased weaning rate compared with MAINT (*P* < 0.01), but did not differ from NP cows (*P* = 0.52). A greater percentage of multiparous cows classified as non-pregnant weaned calves compared with NP primiparous (*P* = 0.01).

**Table 3.** Comparative weaning data between cows that conceived or did not conceive to fixed-time artificial insemination (FTAI) and cows that experienced late embryonic/early fetal mortality (LEEFM).

Item	Pregnancy status^1^				*P*-value		
	Maintained	Non-pregnant	LEEFM	SEM	Status	Parity	Status × Parity
Weaning rate^2^, %					<0.01	0.27	<0.02
Multiparous	93.6^a^	76.1^b,x^	47.5^c^	8.11			
Primiparous	96.0^a^	59.5^b,y^	32.9^b^	13.87			
Weaning weight, kg	253.0^a^	225.7^b^	196.5^c^	15.94	<0.01	<0.01	0.57
205 Adj. weaning weight^3^, kg	231.7	230.2	209.1	13.82	0.11	0.22	0.40
Kg of calf per cow exposed, kg					<0.01	<0.01	<0.01
Multiparous	229.4^a^	176.1^b,x^	95.1^c^	16.82			
Primiparous	221.9^a^	132.8^b,y^	61.2^b^	27.97			

^1^Cows were classified by pregnancy status in year one. MAINT: Maintained FTAI pregnancy; NP: Non-pregnant to FTAI; LEEFM: Late embryonic/early fetal mortality.

^2^Weaning rate was calculated as the percentage of cows that weaned a calf relative to the total number of cows in the herd.

^3^Adjusted weaning weights were  assessed using a 205-day approach, where adjusted weaning weights = birth weight + [205 × (average daily gain between birth and weaning)].

^a,b,c^Within a row represents the statistical difference between pregnancy status (*P* ≤ 0.05).

^x,y^Within a column represents the statistical difference between parity (Multiparous or Primiparous) within the same pregnancy status (*P* ≤ 0.05).

Cows classified as LEEFM weaned lighter calves compared with NP and MAINT cows (*P* < 0.01), whereas NP cows weaned lighter calves compared with MAINT (*P* = 0.01). In addition, multiparous cows weaned heavier (*P* = 0.01) calves compared with primiparous (235.8 ± 13.46 and 214.3 ± 14.36 kg, respectively). There was no interaction between parity and pregnancy status on weaning weight (*P* = 0.57). Pregnancy status (*P *= 0.11), parity (*P = *0.22), and the interaction between pregnancy status and parity (*P *= 0.39) did not impact 205-d adjusted weaning weights.

There was an interaction (*P* = 0.01) between pregnancy status and parity for kg of calf produced per cow exposed. Within multiparous, cows classified as LEEFM weaned less kg of calves per cow exposed compared with NP (*P *= 0.01) and MAINT (*P* = 0.01) cows, whereas MAINT cows produced more kg of calves per cow exposed compared with NP cows (*P* < 0.01). In primiparous, MAINT cows produced more kg of calves compared with NP and LEEFM (*P* < 0.01) cows; however, NP did not differ (*P* = 0.09) from LEEFM. Multiparous cows classified as NP produced more (*P* = 0.01) kg of calves per cow exposed than primiparous cows.

### Pregnancy Outcomes in the Subsequent Breeding Season

**Figure 3.**
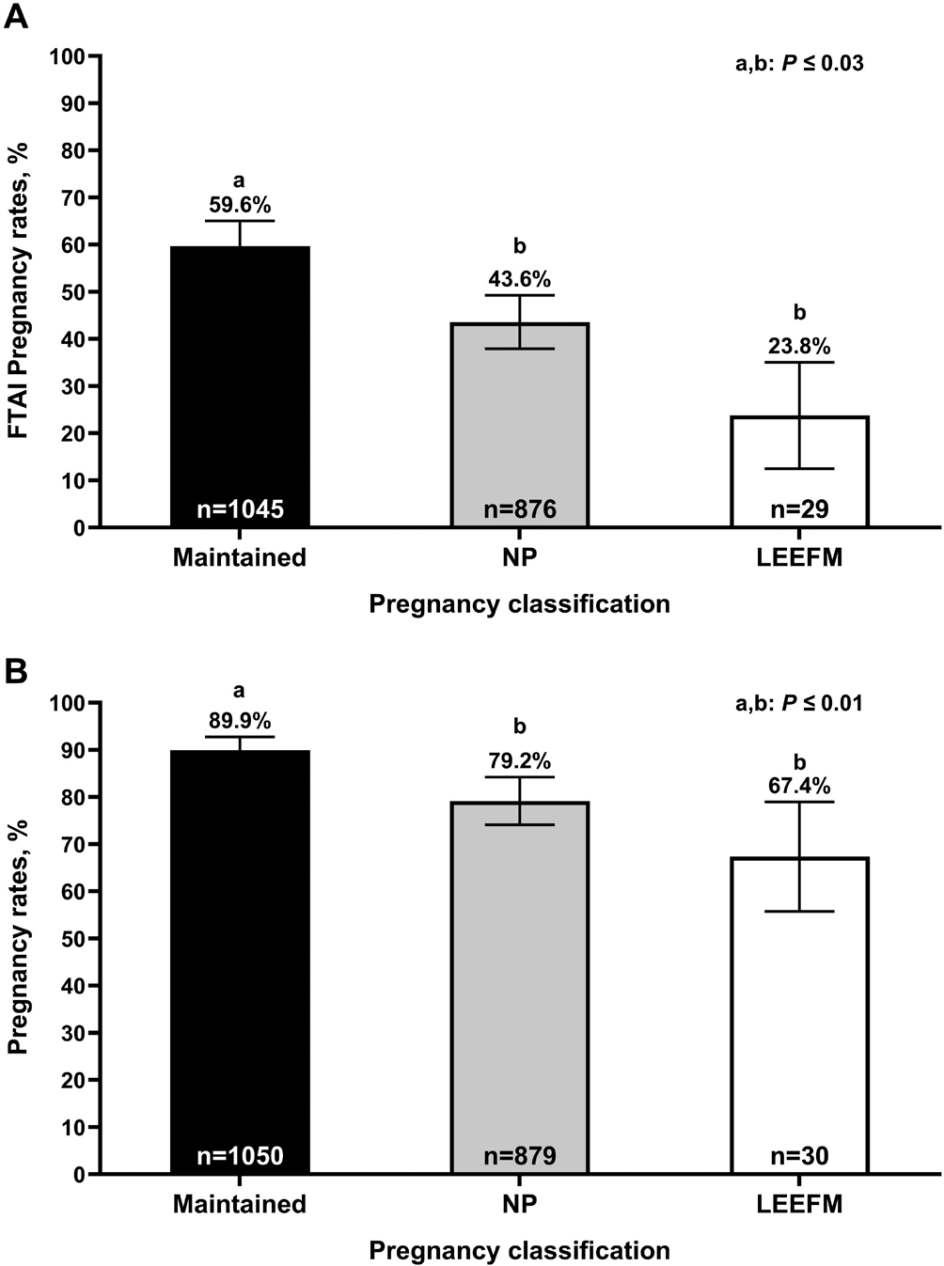
Year two fixed-timed artificial insemination (FTAI; A) and final pregnancy rates (B) in beef cows subjected to FTAI followed by natural service. Cows were classified by pregnancy status in year one. MAINT: Maintained FTAI pregnancy; NP: Non-pregnant to FTAI; LEEFM: Late embryonic/early fetal mortality. ^a,b^ Uncommon superscripts indicate statistical differences (*P* ≤ 0.03).

Figure 3 describes pregnancy outcomes in year two based on year one pregnancy status classification. Cows classified as MAINT had greater pregnancy rates to FTAI (Figure 3A) and final pregnancy rates (Figure 3B) compared with NP and LEEFM (*P* ≤ 0.03), but there was no difference (*P* ≥ 0.28) between NP and LEEFM. There was no effect of parity (*P* = 0.22) or interaction (P = 0.26) between parity and pregnancy status observed for pregnancy rates to FTAI and final pregnancy rates in the second year.

## DISCUSSION

The present study evaluated the impact of late embryonic and early fetal mortality on the overall productivity of postpartum beef cows exposed to one round of FTAI followed by natural service for the remainder of the breeding season. To our knowledge, this is the first large-scale study assessing the effects of LEEFM over an entire production cycle and two consecutive breeding seasons. Thus, our results fill a relevant gap in the current literature and help characterize the production consequences of LEEFM in beef cattle herds.

Pregnancy rates to FTAI and final pregnancy rates in the present study were 50.8 and 88.3%, respectively. The percentage of cows classified as undergoing LEEFM was 2.3%, which represented 5.1% of the cows that conceived to FTAI. The prevalence of LEEFM in the present study was slightly lower than in some studies (Aono et al., 2013) but comparable to other reports in the literature (Dobbins et al., 2009; Sá Filho et al., 2014). This relatively decreased prevalence of LEEFM was likely a consequence of the fact that the herds enrolled in the present study are well-managed operations, with adequate nutritional programs, strict health protocols, and biosecurity measures. Additionally, virgin heifers, which have been reported to have greater LEEFM (Sales et al., 2011), were not included in the present dataset. Late embryonic/early fetal mortality was also only evaluated in cows exposed to FTAI, a breeding method that has been repeatedly shown to result in less LEEFM compared with other assisted reproductive technologies, such as embryo transfer following in vitro embryo production (Munhoz et al., 2024; Prado et al., 2024). Hence, although the prevalence of LEEFM was relatively low in the present study, this prevalence was representative of well managed herds in the United States and also provided enough statistical power to characterize the impact of LEEFM on beef cow productivity.

The present study initially aimed to investigate the impact of LEEFM on year one pregnancy outcomes at the end of the breeding season. Cows that experienced LEEFM experienced a 34.4% decrease in pregnancy rates at the end of the first breeding season compared with cows that did not conceive to FTAI (NP cows). Controlled studies reporting final pregnancy outcomes for beef cows experiencing LEEFM within a short breeding season are limited, and the timing in which cows conceive after undergoing LEEFM during natural service breeding is not well characterized. Beef cattle producers that manage cows in a short breeding season typically expose females to clean-up bulls for approximately 45 to 90 d following FTAI (Boyer et al., 2020), providing them two to four consecutive estrous cycles to become pregnant before bull removal (Deutscher et al., 1991). In the present study, the length of the breeding seasons ranged from 67 to 86 d across different herds, which resembled industry practices and resulted in a relatively short period for LEEFM cows to conceive after undergoing pregnancy loss. Therefore, it is unsurprising that pregnancy rates at the end of the breeding season for LEEFM were not only decreased compared with cows that maintained their pregnancy but also compared with cows that failed to conceive to FTAI.

Differences in final pregnancy rates between cohorts resulted in LEEFM cows having reduced calving and weaning rates compared with cows that maintained their FTAI pregnancy and cows that failed to conceive to FTAI. Moreover, our results demonstrate clear differences between cohorts in calving distribution. LEEFM cows calved later in the calving season when compared with MAINT and NP cows. In fact, more than 70% of LEEFM cows calved during the second half of the calving season, resulting in an average calving day of 51 d. These results are attributed to the fact that LEEFM cows conceived at least one month later than MAINT cows, as pregnancy loss in LEEFM cows occurred no earlier than 28 d after FTAI (Diskin and Morris, 2008; Wiltbank et al., 2016; Hansen, 2020). Most of the cows classified as non-pregnant to FTAI calved in the second period of the calving season, leading to a decreased average calving day compared to LEEFM cows. Timlin et al. (2021) reported that 51% of cows that failed to conceive to FTAI became pregnant by natural service on their subsequent estrus (approximately 21 d after FTAI), which supports our findings that approximately 67% of NP cows calved within the first 42 d. As expected, primiparous cows classified as NP calved 5.7 d later than multiparous cows classified as NP. Hence, primiparous cows that failed to conceive to FTAI likely conceived later in the breeding season compared with NP multiparous cows. Primiparous cows often face a more pronounced negative energy balance due to the combined energy demands for growth and lactation, leading to a greater proportion of females in anestrous at the beginning of the breeding season (Stevenson et al., 2015; Ferreira et al., 2021).

Calves from cows classified as LEEFM were 29.2 kg lighter than those from NP cows and 56.5 kg lighter than those from MAINT cows at weaning. Funston et al. (2012) reported that calves born towards the end of the calving season present decreased weaning weights compared with calves born earlier, despite similar average daily gains between birth and weaning. This corroborates with the present findings that calves from LEEFM cows, which were born later in the calving season, had decreased weaning weights compared with calves from MAINT and NP cows. Although age at weaning is a major driver of weaning weights, other factors could have contributed to the decreased weaning weights observed in calves from LEEFM cows, such as genetic superiority for pre-weaning growth in FTAI calves (Leighton et al., 1982). To account for the impact of calf age on weaning weights, a 205-d weaning weight adjustment was performed. No differences in 205-d adjusted weaning weights were observed between pregnancy status classifications. This indicates that the reduced weaning weights in calves from LEEFM cows in this present study was primarily attributed to their late conception and delayed calving (Funston et al., 2012). These weaning weight differences, combined with differences in weaning rates between cohorts, resulted in fewer kg of calf produced per cow exposed to the breeding season in the LEEFM cohort.

Pregnancy outcomes in the second year were also assessed to evaluate the long-term impact of LEEFM on cow fertility. Approximately 60% of cows that conceived to FTAI and maintained their pregnancy after the year one breeding season also conceived to FTAI in the second year. Moreover, MAINT cows had greater FTAI and final pregnancy rates in year two compared with both NP and LEEFM cows. No significant differences were observed between NP and LEEFM for both FTAI and final pregnancy rates in year two. Yet, it is worth noting that the LEEFM cohort had a limited number of observations (n = 30) in year two, which may have hindered our ability to detect statistical differences between these cohorts.

Calving distribution is a major driver of subsequent pregnancy rates in beef cow-calf production systems. Cows that calve early in the calving season have more time to recover from parturition before the beginning of the subsequent breeding season, allowing early calving cows to resume postpartum cyclicity prior to the next breeding season, which increases their likelihood of becoming pregnant (Stevenson et al., 2003, 2015; Cushman et al., 2013). Our data indicates that LEEFM and NP cows calved later in the calving season when compared with MAINT cows, resulting in a shorter postpartum recovery period prior to the subsequent breeding season. Delayed calving in NP and LEEFM cows was, therefore, likely a major contributor to their suboptimal fertility in year two. Alternatively, it is also reasonable to speculate that differences in year two fertility are partially associated with MAINT cows having intrinsically greater fertility compared with NP and LEEFM (Geary et al., 2016).

In summary, approximately 60% of cows that experienced LEEFM failed to conceive within the same breeding season. Consequently, LEEFM cows had reduced calving and weaning rates in the following year compared with both MAINT and NP cows. Among LEEFM cows that did conceive, pregnancy occurred later in the breeding season compared with MAINT and NP cows. As a result, calving distribution in the subsequent year was altered, with LEEFM cows calving later than their counterparts. Moreover, delayed calving in LEEFM cows resulted in reduced weaning weights and suboptimal fertility in the following year. To our knowledge, this study is the first to thoroughly characterize the long-term impact of LEEFM across a complete production cycle in beef cattle, providing valuable insights into its effects on reproductive performance and overall cow productivity. Moreover, these findings highlight the detrimental impact of LEEFM on cow production efficiency and underscore the importance of strategies to minimize pregnancy losses.

## References

[CIT0001] Andersen, C. M., R. C. Bonacker, E. G. Smith, C. M. Spinka, S. E. Poock, and J. M. Thomas. 2021. Evaluation of the 7 & 7 Synch and 7-day CO-Synch + CIDR treatment regimens for control of the estrous cycle among beef cows prior to fixed-time artificial insemination with conventional or sex-sorted semen. Anim. Reprod. Sci. 235:106892. doi: https://doi.org/10.1016/j.anireprosci.2021.106892 34861592

[CIT0002] Aono, F. H., R. F. Cooke, A. A. Alfieri, and J. L. M. Vasconcelos. 2013. Effects of vaccination against reproductive diseases on reproductive performance of beef cows submitted to fixed-timed AI in Brazilian cow-calf operations. Theriogenology. 79:242–248. doi: https://doi.org/10.1016/j.theriogenology.2012.08.008 23174768

[CIT0003] Boyer, C., A. Griffith, and K. Pohler. 2020. Improving beef cattle profitability by changing calving season length. J. Appl. Farm Econ. 3:19–30. doi: https://doi.org/10.7771/2331-9151.1035. https://docs.lib.purdue.edu/jafe/vol3/iss1/2

[CIT0004] Brandão, A. P., and R. F. Cooke. 2021. Effects of temperament on the reproduction of beef cattle. Animals. 11:3325. doi: https://doi.org/10.3390/ani11113325 34828056 PMC8614566

[CIT0005] Consentini, C. E. C., R. L. O. R. Alves, M. A. Silva, J. P. A. Galindez, G. Madureira, L. G. Lima, J. R. S. Gonçalves, M. C. Wiltbank, and R. Sartori. 2023. What are the factors associated with pregnancy loss after timed-artificial insemination in Bos indicus cattle? Theriogenology. 196:264–269. doi: https://doi.org/10.1016/j.theriogenology.2022.10.037 36436362

[CIT0006] Credille, B. C., J. D. Duggin, A. L. Jones, G. Nyhuis, P. L. P. Fontes, R. L. Stewart, and R. D. Berghaus. 2023. Physical traits, performance data, and reproductive tract maturity score can be used to predict fertility and likelihood of early conception in beef replacement heifers consigned to a heifer development program in the southeastern United States. J Am Vet Med Assoc. 261:1193–1199. doi: https://doi.org/10.2460/javma.23.02.0093 37059423

[CIT0007] Cushman, R. A., L. K. Kill, R. N. Funston, E. M. Mousel, and G. A. Perry. 2013. Heifer calving date positively influences calf weaning weights through six parturitions1. J. Anim. Sci. 91:4486–4491. doi: https://doi.org/10.2527/jas.2013-6465 23825337

[CIT0008] Deutscher, G. H., J. A. Stotts, and M. K. Nielsen. 1991. Effects of breeding season length and calving season on range beef cow productivity. J. Anim. Sci. 69:3453–3460. doi: https://doi.org/10.2527/1991.6993453x 1938632

[CIT0009] Dill, M. D., G. R. Pereira, J. B. G. Costa, L. C. Canellas, V. Peripolli, J. B. Neto, D. M. Sant’Anna, C. McManus, and J. O. J. Barcellos. 2015. Technologies that affect the weaning rate in beef cattle production systems. Trop. Anim. Health Prod. 47:1255–1260. doi: https://doi.org/10.1007/s11250-015-0856-x 26048693

[CIT0010] Diskin, M., and D. Morris. 2008. Embryonic and early foetal losses in cattle and other ruminants. Reprod. Domest. Anim. 43:260–267. doi: https://doi.org/10.1111/j.1439-0531.2008.01171.x 18638133

[CIT0037] Dobbins, C.A., Eborn, D.R., Tenhouse, D.E., Breiner, R.M., Johnson, S.K., Marston, T.T., Stevenson, J.S. 2009. Insemination timing affects pregnancy rates in beef cows treated with CO-Synch protocol including an intravaginal progesterone insert. Theriogenology. 72:1009-1016. 19726074 10.1016/j.theriogenology.2009.06.025

[CIT0011] Ealy, A. D. 2020. Pregnancy losses in livestock: an overview of the physiology and endocrinology symposium for the 2020 ASAS-CSAS-WSASAS Virtual Meeting. J. Anim. Sci. 98:skaa277. doi: https://doi.org/10.1093/jas/skaa277 32931570 PMC7491922

[CIT0012] Ealy, A. D., L. K. Wooldridge, and S. R. McCoski. 2019. BOARD INVITED REVIEW: post-transfer consequences of in vitro-produced embryos in cattle. J. Anim. Sci. 97:2555–2568. doi: https://doi.org/10.1093/jas/skz116 30968113 PMC6541818

[CIT0013] Ferreira, M. F. de L., L. N. Rennó, I. I. Rodrigues, E. Detmann, M. F. Paulino, S. de Campos Valadares Filho, H. C. Martins, S. S. Moreira, and D. S. de Lana. 2021. Effects of parity order on performance, metabolic, and hormonal parameters of grazing beef cows during pre-calving and lactation periods. BMC Vet. Res. 17:311. doi: https://doi.org/10.1186/s12917-021-03019-0 34563192 PMC8467019

[CIT0014] Fontes, P. L. P., R. F. Cooke, N. Oosthuizen, C. L. Timlin, N. W. Dias, J. F. Currin, S. Clark, K. G. Pohler, G. C. Lamb, and V. R. G. Mercadante. 2019. Impacts of administering prostaglandin F2α analogue 24 h prior to progesterone insert removal on expression of estrus in beef females. Livest. Sci. 226:82–86. doi: https://doi.org/10.1016/j.livsci.2019.06.007

[CIT0015] Funston, R. N., J. A. Musgrave, T. L. Meyer, and D. M. Larson. 2012. Effect of calving distribution on beef cattle progeny performance. J. Anim. Sci. 90:5118–5121. doi: https://doi.org/10.2527/jas.2012-5263 22871928

[CIT0016] Geary, T. W., G. W. Burns, J. G. N. Moraes, J. I. Moss, A. C. Denicol, K. B. Dobbs, M. S. Ortega, P. J. Hansen, M. E. Wehrman, H. Neibergs, et al 2016. Identification of beef heifers with superior uterine capacity for pregnancy. Biol. Reprod. 95:47–47. doi: https://doi.org/10.1095/biolreprod.116.141390 27417907 PMC5029478

[CIT0017] Hansen, P. J. 2020. The incompletely fulfilled promise of embryo transfer in cattle—why aren’t pregnancy rates greater and what can we do about it? J. Anim. Sci. 98:skaa288. doi: https://doi.org/10.1093/jas/skaa288 33141879 PMC7608916

[CIT0018] Koziol, J. H., and C. L. Armstrong. 2018. Manual for breeding soundness examination of bulls. 4th ed. Montgomery, AL: Society for Theriogenology.

[CIT0019] Larson, J. E., G. C. Lamb, J. S. Stevenson, S. K. Johnson, M. L. Day, T. W. Geary, D. J. Kesler, J. M. DeJarnette, F. N. Schrick, A. DiCostanzo, et al 2006. Synchronization of estrus in suckled beef cows for detected estrus and artificial insemination and timed artificial insemination using gonadotropin-releasing hormone, prostaglandin F2α, and progesterone1. J. Anim. Sci. 84:332–342. doi: https://doi.org/10.2527/2006.842332x 16424261

[CIT0020] Leighton, E. A., R. L. Willham, and P. J. Berger. 1982. Factors influencing weaning weight in the hereford cattle and adjustment factors to correct records for these effects. J. Anim. Sci. 54:957–963. doi: https://doi.org/10.2527/jas1982.545957x

[CIT0021] Mercadante, V. R. G., N. W. Dias, C. L. Timlin, and S. Pancini. 2020. 375 Economic Consequences of pregnancy loss in Beef Cattle. J. Anim. Sci. 98:124–124. doi: https://doi.org/10.1093/jas/skaa278.226

[CIT0022] Munhoz, S. K., R. F. Cooke, A. K. Munhoz, C. P. Prado, M. H. C. Pereira, and J. L. M. Vasconcelos. 2024. Pregnancy losses in *Bos indicus-*influenced beef and dairy recipients assigned to a fixed-time embryo transfer protocol. Anim. Reprod. Sci. 264:107471. doi: https://doi.org/10.1016/j.anireprosci.2024.107471 38581821

[CIT0023] Oosthuizen, N., P. L. P. Fontes, K. Porter, and G. C. Lamb. 2020. Presynchronization with prostaglandin F2α and prolonged exposure to exogenous progesterone impacts estrus expression and fertility in beef heifers. Theriogenology. 146:88–93. doi: https://doi.org/10.1016/j.theriogenology.2020.02.010 32062494

[CIT0024] Pereira, M. H. C., M. C. Wiltbank, and J. L. M. Vasconcelos. 2016. Expression of estrus improves fertility and decreases pregnancy losses in lactating dairy cows that receive artificial insemination or embryo transfer. J. Dairy Sci. 99:2237–2247. doi: https://doi.org/10.3168/jds.2015-9903 26723130

[CIT0025] Prado, C. P., R. F. Cooke, A. K. Munhoz, S. K. Munhoz, M. C. G. de Sousa, V. M. P. da Silva, K. G. Pohler, and J. L. M. Vasconcelos. 2024. Characterizing pregnancy losses in *Bos indicus* beef females receiving a fixed-timed artificial insemination protocol. Theriogenology 215:144–150. doi: https://doi.org/10.1016/j.theriogenology.2023.11.013 38070213

[CIT0026] Ramsey, R., D. Doye, C. Ward, J. McGrann, L. Falconer, and S. Bevers. 2005. Factors affecting beef cow-herd costs, production, and profits. J. Agric. Appl. Econ. 37:91–99. doi: https://doi.org/10.1017/s1074070800007124

[CIT0027] Reese, S. T., G. A. Franco, R. K. Poole, R. Hood, L. Fernadez Montero, R. V. Oliveira Filho, R. F. Cooke, and K. G. Pohler. 2020. Pregnancy loss in beef cattle: a meta-analysis. Anim. Reprod. Sci. 212:106251. doi: https://doi.org/10.1016/j.anireprosci.2019.106251 31864492

[CIT0038] Sá Filho, M.F. , Marques, M.O., Girotto, R., Santos, F.A., Sala, R.V., Barbuio, J.P., Baruselli, P.S. 2014. Resynchronization with unknown pregnancy status using progestin-based timed artificial insemination protocol in beef cattle. Theriogenology. 81:284-290. doi: https://doi.org/10.1016/j.theriogenology.2013.09.027. 24139935

[CIT0028] Sales, J. N. S., R. V. V. Pereira, R. C. Bicalho, and P. S. Baruselli. 2011. Effect of injectable copper, selenium, zinc and manganese on the pregnancy rate of crossbred heifers (*Bos indicus* × *Bos taurus*) synchronized for timed embryo transfer. Livest. Sci. 142:59–62. doi: https://doi.org/10.1016/j.livsci.2011.06.014

[CIT0029] Sanhueza, J. M., C. Heuer, and D. West. 2013. Contribution of Leptospira, Neospora caninum and bovine viral diarrhea virus to fetal loss of beef cattle in New Zealand. Prev. Vet. Med. 112:90–98. doi: https://doi.org/10.1016/j.prevetmed.2013.07.009 23932894

[CIT0030] Santos, J. E. P., W. W. Thatcher, R. C. Chebel, R. L. A. Cerri, and K. N. Galvão. 2004. The effect of embryonic death rates in cattle on the efficacy of estrus synchronization programs. Anim. Reprod. Sci. 82-83:513–535. doi: https://doi.org/10.1016/j.anireprosci.2004.04.015 15271477

[CIT0031] Smith, B. D., B. Poliakiwski, O. Polanco, S. Singleton, G. D. de Melo, M. Muntari, R. V. O. Filho, and K. G. Pohler. 2022. Decisive points for pregnancy losses in beef cattle. Reprod. Fertil. Dev. 35:70–83. doi: https://doi.org/10.1071/RD22206 36592980

[CIT0032] Stevenson, J. S., S. L. Hill, G. A. Bridges, J. E. Larson, and G. C. Lamb. 2015. Progesterone status, parity, body condition, and days postpartum before estrus or ovulation synchronization in suckled beef cattle influence artificial insemination pregnancy outcomes1. J. Anim. Sci. 93:2111–2123. doi: https://doi.org/10.2527/jas.2014-8391 26020307

[CIT0033] Stevenson, J. S., G. C. Lamb, S. K. Johnson, M. A. Medina-Britos, D. M. Grieger, K. R. Harmoney, J. A. Cartmill, S. Z. El-Zarkouny, C. R. Dahlen, and T. J. Marple. 2003. Supplemental norgestomet, progesterone, or melengestrol acetate increases pregnancy rates in suckled beef cows after timed inseminations1. J. Anim. Sci. 81:571–586. doi: https://doi.org/10.2527/2003.813571x 12661636

[CIT0034] Timlin, C. L., N. W. Dias, L. Hungerford, T. Redifer, J. F. Currin, and V. R. G. Mercadante. 2021. A retrospective analysis of bull:cow ratio effects on pregnancy rates of beef cows previously enrolled in fixed-time artificial insemination protocols. Transl Anim Sci 5:txab129. doi: https://doi.org/10.1093/tas/txab129 34514347 PMC8427174

[CIT0035] Wagner, J. J., K. S. Lusby, J. W. Oltjen, J. Rakestraw, R. P. Wettemann, and L. E. Walters. 1988. Carcass composition in mature hereford cows: estimation and effect on daily metabolizable energy requirement during winter. J. Anim. Sci. 66:603–612. doi: https://doi.org/10.2527/jas1988.663603x 3378920

[CIT0036] Wiltbank, M. C., G. M. Baez, A. Garcia-Guerra, M. Z. Toledo, P. L. J. Monteiro, L. F. Melo, J. C. Ochoa, J. E. P. Santos, and R. Sartori. 2016. Pivotal periods for pregnancy loss during the first trimester of gestation in lactating dairy cows. Theriogenology. 86:239–253. doi: https://doi.org/10.1016/j.theriogenology.2016.04.037 27238438

